# Sex differences in the risk of vascular disease associated with diabetes

**DOI:** 10.1186/s13293-019-0277-z

**Published:** 2020-01-03

**Authors:** Rianneke de Ritter, Marit de Jong, Rimke C. Vos, Carla J. H. van der Kallen, Simone J. S. Sep, Mark Woodward, Coen D. A. Stehouwer, Michiel L. Bots, Sanne A. E. Peters

**Affiliations:** 1 Department of Internal Medicine, Maastricht University Medical Centre+ , Maastricht, The Netherlands; 2 CARIM Cardiovascular Research Institute Maastricht, Maastricht University, Maastricht, The Netherlands; 3 Julius Center for Health Sciences and Primary Care, University Medical Center Utrecht, Utrecht University, Utrecht, The Netherlands; 4 Department Public Health and Primary Care / LUMC-Campus The Hague, Leiden University Medical Center, The Hague, The Netherlands; 5 The George Institute for Global Health, University of Oxford, Oxford, UK; 6 The George Institute for Global Health, University of New South Wales, Sydney, Australia; 7 Department of Epidemiology, Johns Hopkins University, Baltimore, Maryland USA

## Abstract

Diabetes is a strong risk factor for vascular disease. There is compelling evidence that the relative risk of vascular disease associated with diabetes is substantially higher in women than men. The mechanisms that explain the sex difference have not been identified. However, this excess risk could be due to certain underlying biological differences between women and men. In addition to other cardiometabolic pathways, sex differences in body anthropometry and patterns of storage of adipose tissue may be of particular importance in explaining the sex differences in the relative risk of diabetes-associated vascular diseases. Besides biological factors, differences in the uptake and provision of health care could also play a role in women’s greater excess risk of diabetic vascular complications. In this review, we will discuss the current knowledge regarding sex differences in both biological factors, with a specific focus on sex differences adipose tissue, and in health care provided for the prevention, management, and treatment of diabetes and its vascular complications. While progress has been made towards understanding the underlying mechanisms of women’s higher relative risk of diabetic vascular complications, many uncertainties remain. Future research to understanding these mechanisms could contribute to more awareness of the sex-specific risk factors and could eventually lead to more personalized diabetes care. This will ensure that women are not affected by diabetes to a greater extent and will help to diminish the burden in both women and men.

## Background

Diabetes is one of the most common chronic diseases globally. In 2017, an estimated 425 million adults, 8.4% of women and 9.1% of men, had diabetes, and an additional 352 million adults were at risk of developing the condition [1]. The prevalence of diabetes is expected to further rise by 48%, to 629 million affected adults aged between 20 and 79 years by 2045 [1]. The two main types of diabetes are diabetes type 1 and diabetes type 2, accounting for ~ 5–10% and ~ 90% of all individuals with diabetes, respectively [1, 2]. Although diabetes type 2 is most often diagnosed at middle or old age, it is increasingly common in children, adolescents, and young adults, often as a consequence of obesity, physical inactivity, and poor dietary habits [1, 3].

Diabetes is a major contributor to premature mortality. In 2017, an estimated 4 million deaths of people aged between 20 and 79 years were attributed to diabetes [1], making it the seventh most common cause of death worldwide [4]. More women than men die of diabetes on a global scale: 2.1 versus 1.8 million in 2017 [1]. The only regions where more men than women die from diabetes are North America and the Caribbean region [1]. Individuals with diabetes are at increased risk of cardiovascular complications, chronic kidney disease, certain cancers, physical and cognitive impairment (i.e., dementia), depression, and respiratory and other infectious diseases [1, 5, 6].

Cardiovascular disease is the most common complication of diabetes and can be broadly categorized in microvascular complications (classically, neuropathy, nephropathy, and retinopathy) and macrovascular complications including coronary artery disease, stroke, and peripheral arterial disease. Individuals with diabetes are two to three times more likely to develop cardiovascular disease compared to individuals without diabetes [1].

However, not everyone with diabetes has the same excess risk of cardiovascular disease. Large-scale systematic reviews with meta-analyses have demonstrated that the excess risk of macrovascular complications associated with diabetes is substantially greater in women than men [7, 8]. The relative risks of incident coronary heart disease (CHD) and stroke, respectively, associated with diabetes have been estimated to be 44% and 27% higher in women than men [7, 8]. Likewise, another meta-analysis of 68 prospective studies has shown that, after adjustment for major vascular risk factors, diabetes was associated with a nearly 50% higher occlusive vascular mortality rate among women than men [9]. The excess risk of vascular mortality among women conferred by diabetes was especially high among those between the age of 35 and 59 years, with almost a six times higher occlusive vascular death rate among women and a nearly two and a half times higher rate among men [9]. Another meta-analysis demonstrated that diabetes was associated with a 19% higher relative risk of vascular dementia in women than men [10]. A sex differential in the consequences of diabetes has also been shown for end stage renal disease, where the relative risk of end-stage renal disease was 38% higher among women than men [11]. Since 90% of individuals with diabetes have type 2 diabetes, most individuals with diabetes who were included in these meta-analyses had type 2 diabetes. Nevertheless, a meta-analysis that specifically focused on type 1 diabetes has shown that women with type 1 diabetes had almost a 40% higher relative risk of all-cause mortality, and a 200% higher relative risk of fatal and nonfatal vascular events, compared with men with type 1 diabetes [12].

**Fig. 1:**
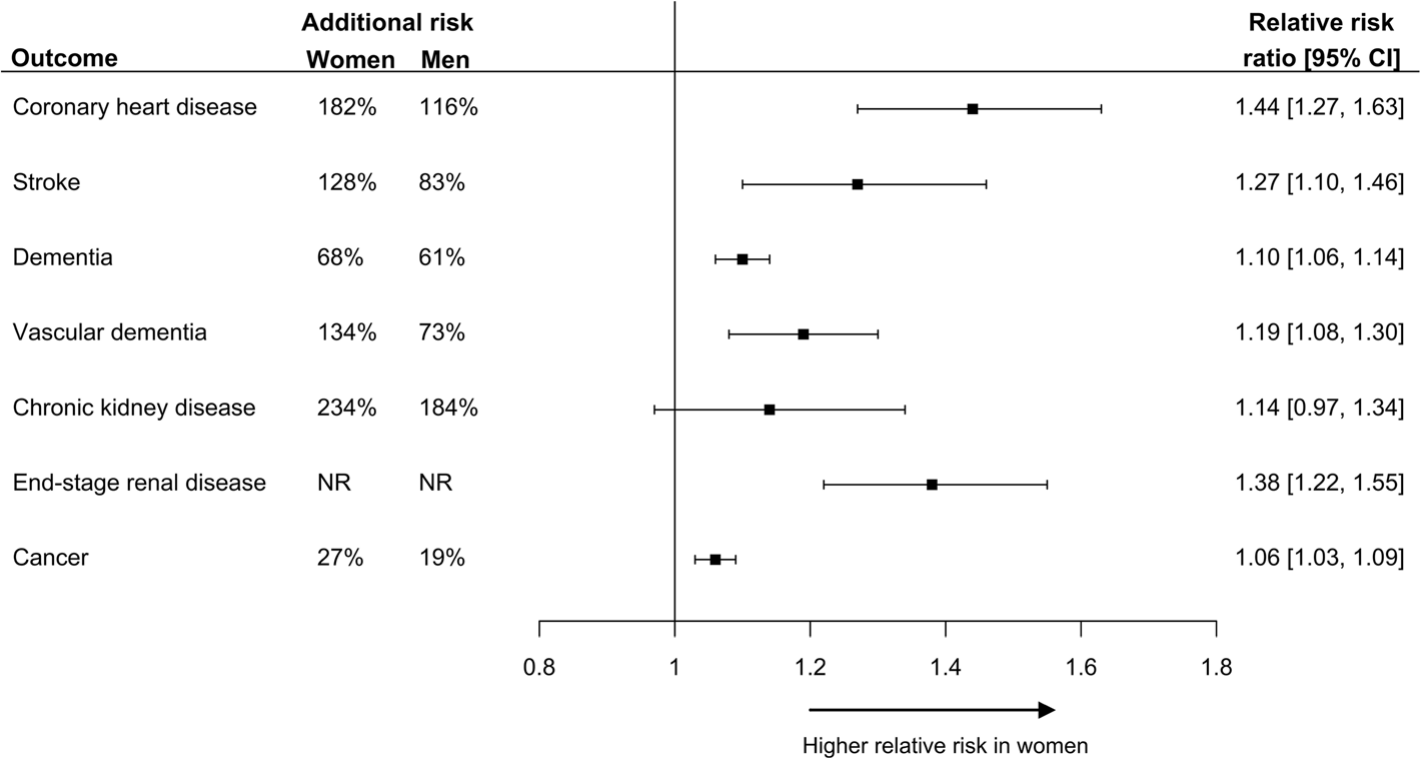
Results from prior meta-analyses of sex differences in the effects of diabetes on vascular outcomes and cancer expressed as the women-to-men ratio of relative risks (RRR) and the additional risks [7, 8, 10, 11, 13]. RRR, relative risk ratio; RR, relative risk; NR, not reported

In addition to vascular disease, sex differences may also exist in the association between diabetes and non-vascular diseases. A recent meta-analysis has shown that women have a 6% greater relative risk of diabetes-associated cancer, with some variation by cancer type [13]. Sex differences in other non-vascular diseases require further study. Figure 1 summarizes the results from the abovementioned meta-analyses.

While the greater excess risk of vascular complications conferred by diabetes in women compared with men has been well described, mechanisms underpinning the sex difference have not been identified in full. In this review, we will first discuss sex differences in biological factors, with a specific focus on adipose tissue, and secondly, we will discuss sex differences in the uptake and provision of health care. These mechanisms may be involved in explaining the sex difference in the vascular consequences of diabetes. Although some aspects may differ by type of diabetes, we shall mainly focus on diabetes in general, while acknowledging that most cases with diabetes would have type 2 diabetes.

### Biological aspects

Women and men are subject to similar environmental exposures during their life course, but they are biologically different. For that reason, the excess risk of diabetes-associated vascular disease in women compared with men could be due to physiological, such as hormonal or genetic, differences between women and men.

To diagnose diabetes, an arbitrary cutoff value of a continuous trait is used, such as fasting blood glucose (FG) or glycated hemoglobin (HbA1c). Nevertheless, there is compelling evidence of a progressive association between various measures of glycemia and the risk of vascular disease, both above and below the clinical threshold for diabetes. It has been postulated that, compared with men, metabolic risk factors in women has to deteriorate to a greater magnitude across this continuous trait for diabetes to develop [8, 14]. As a consequence, the exposure to a hazardous cardiometabolic environment in the development of diabetes may be more pronounced in women [8, 15]. This hypothesis is supported by a study that found that, on average, men have prediabetes for 8.5 years and women for 10.3 years prior to the development of diabetes [16]. Moreover, several studies have found a relatively greater increase in the levels of cardiovascular risk factors, in women with diabetes compared with women without diabetes, opposed to their male counterparts [17–20]. Additional to the different impact of risk factors, sex differences in vascular and hormonal pathophysiology could partially explain women’s higher relative risk on diabetes-associated vascular diseases [21]. These potential explanations will be outlined in the next paragraphs.

#### Diabetes-associated sex differences in adiposity

Sex differences in body anthropometry and patterns of storage of adipose tissue may be of particular importance in explaining the sex differences in the diabetes-associated risk of vascular disease [22]. Among 500,000 individuals of the UK Biobank, waist circumference and body mass index (BMI) differed more between women with and without diabetes than between men with and without diabetes [23]. Moreover, when first diagnosed with diabetes, women have a BMI that is nearly 2 kg/m^2^ higher than that of men, despite similar levels of HbA1c [24, 25]. These sex differences in anthropometric characteristics among those with and without diabetes may be linked to differential patterns of fat storage in adipose tissue in women and men [22].

**Fig. 2:**
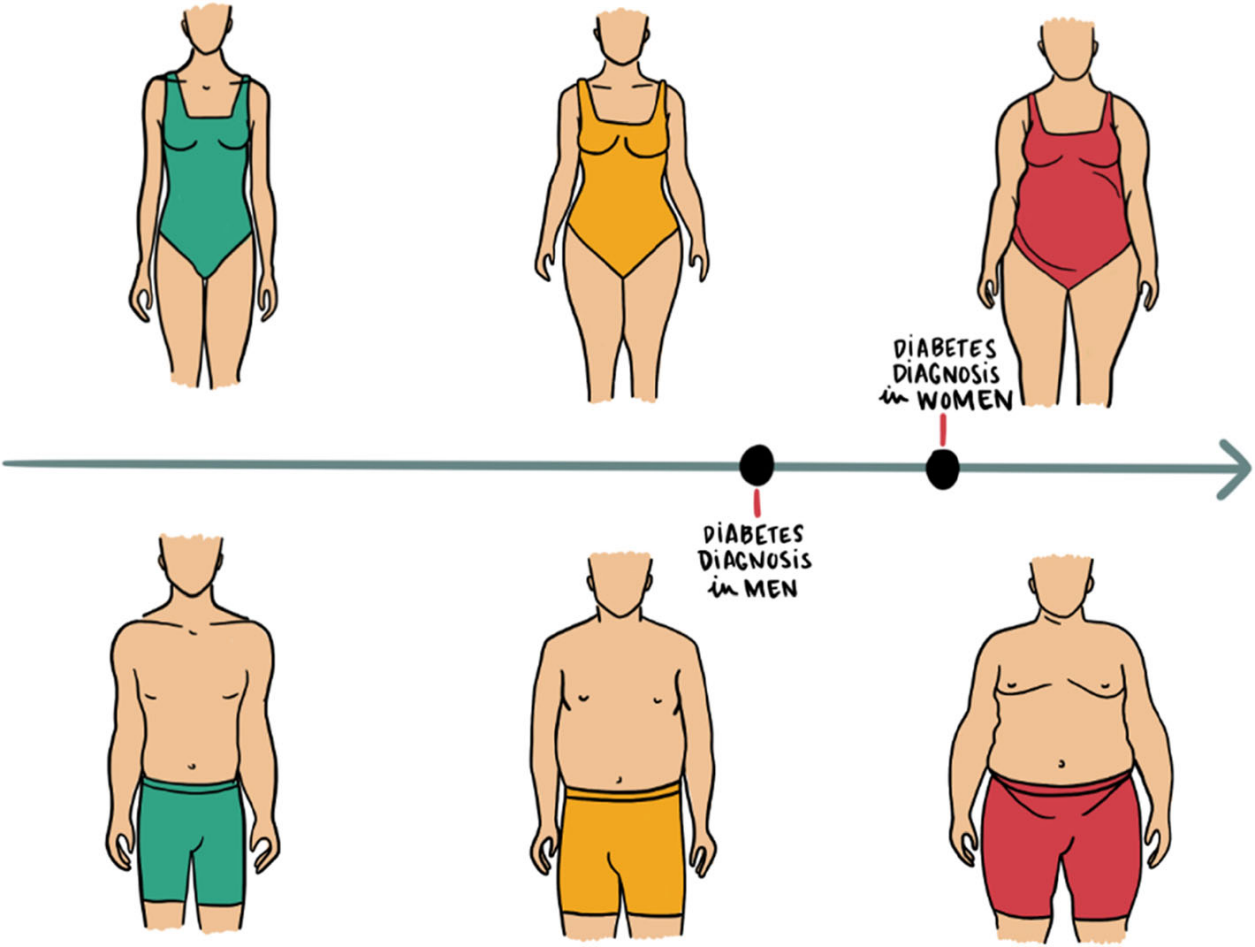
Sex differences in visceral and subcutaneous fat and their association with the time of diagnosis of diabetes

**Fig. 3:**
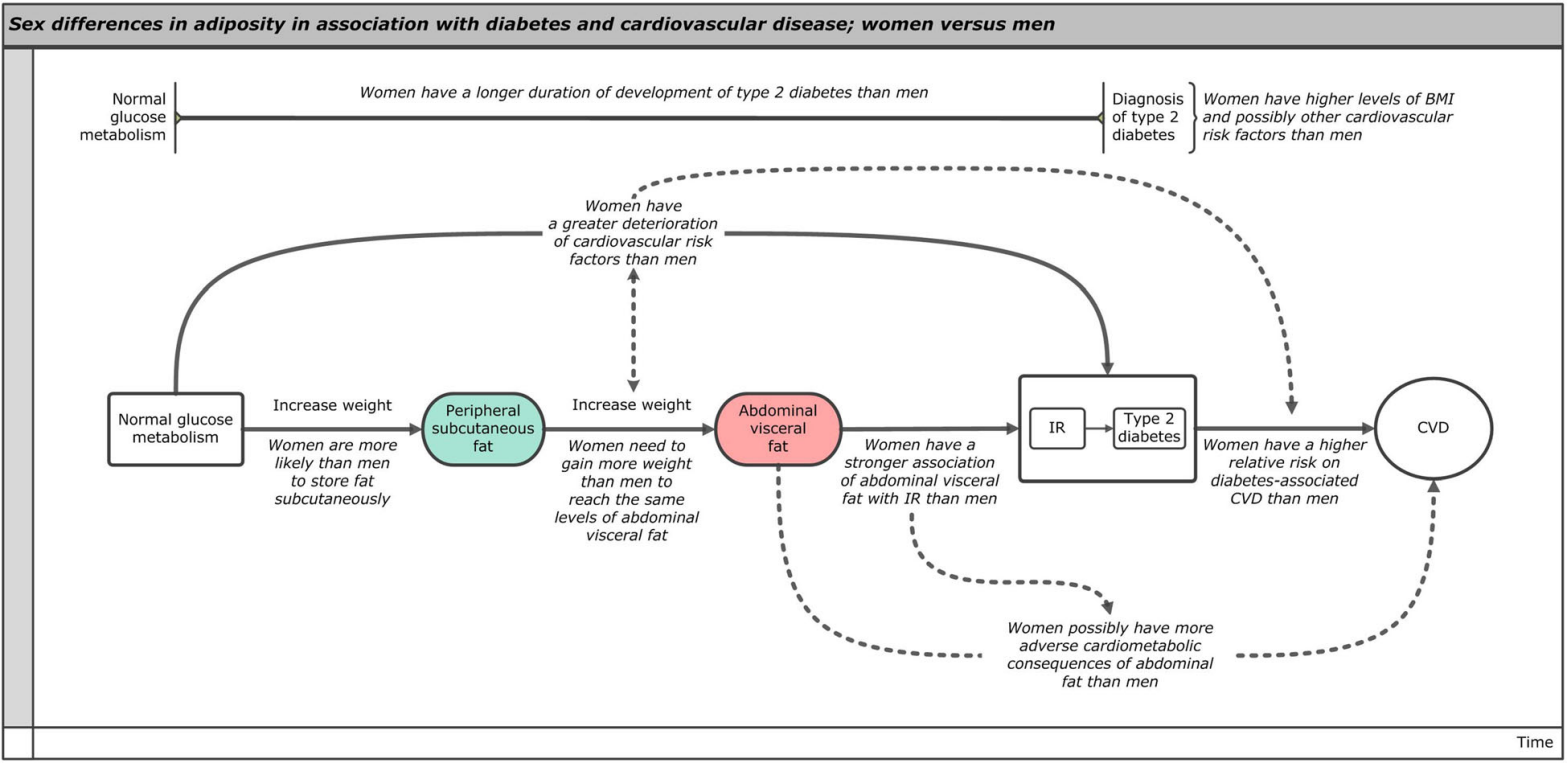
Sex differences in adiposity in association with diabetes and cardiovascular disease. The figure illustrates the associations between adiposity, insulin resistance, type 2 diabetes, and cardiovascular disease in women compared with men. BMI, body mass index; IR, insulin resistance; CVD, cardiovascular disease

Ample evidence exists to show that excess adipose tissue is causally linked to the development of type 2 diabetes and vascular disease [26, 27]. However, it is becoming increasingly apparent that adipose tissue in different parts of the body has different biochemical profiles. In contrast to (peripheral) subcutaneous fat, excess visceral fat and fat in ectopic tissues, like skeletal muscle and the liver, has specifically been associated with insulin resistance [28–30]. This interferes with insulin signaling pathways, which eventually could lead to diabetes [28–30]. Sex differences in the preferred location of fat storage could have an effect on the duration of the development of insulin resistance and diabetes and the consequent deterioration of other related cardiometabolic risk factors. This process is illustrated in Figs. 2 and 3. Women are more likely to store fat subcutaneously and on their lower extremities, whereas men are more likely to store fat in the abdominal region [31]. Correspondingly, men have a substantially higher amount of visceral and ectopic fat compared with premenopausal women, independent of BMI and the amount of total body fat [32, 33]. The preferential deposition of excess fat in visceral and ectopic tissues in men could lead to a faster transition to insulin resistance and diabetes, whereas women may need to gain more weight and related metabolic risk factors might need to deteriorate to a greater extent than in men to reach the same levels of visceral and ectopic fat that are required to develop insulin resistance and eventually diabetes (Fig. 3) [34, 35].

Next to the different metabolic effects of adipose tissue in different parts of the body, abdominal visceral adipose tissue itself seems to have a stronger association with insulin resistance in women than in men, suggesting that excess visceral adipose tissue is more strongly linked to diabetes in women than in men [36]. Likewise, recent findings from the UK Biobank demonstrated that higher waist circumferences and waist-to-hip ratio conferred a greater excess risk of myocardial infarction in women than in men [34]. These findings suggest that excess adipose tissue in the abdominal region may have more adverse cardiometabolic consequences in women than men, which may be explained by sex difference in insulin resistance at a given amount of adipose tissue (Fig. 3).

Finally, there is compelling evidence that obesity and its associated metabolic dysfunction suppresses women’s protective effect of sex-hormones on cardiovascular disease [37]. Adipocytes overfilled with lipids release leptin, which can promote activation of the sympathetic nervous system and the renin-angiotensin system and could stimulate the secretion of aldosterone [38]. In turn, aldosterone is associated with excessive mineralocorticoid receptor signaling on endothelial cells, which play a major role in obesity-associated cardiovascular disease [37, 38]. Women may be predisposed to heightened endothelial mineralocorticoid receptor activation. This might be explained by higher endogenous expression of endothelial mineralocorticoid receptors in blood vessels in women than in men, possibly driven by progesterone receptor activation in in endothelial cells [37]. Moreover, these disadvantageous obesity-associated mechanisms in women may be stronger in the presence of type 2 diabetes, since women have a higher BMI and subsequently more adipose tissue at moment of diagnosis of diabetes than men [24, 25].

#### Diabetes-associated sex differences in other cardiovascular risk factors and vascular pathophysiology

As previously mentioned, it has been hypothesized that women have to undergo greater metabolic deterioration to develop diabetes than men. This hypothesis is also supported by studies that found that sex differences in metabolic risk factors already occur in the transition from normoglycemia to elevated glucose levels and diabetes [39, 40]. During 8 years of follow-up, women who converted to diabetes showed relatively worse levels of total cholesterol, HDL cholesterol, triglycerides, and diastolic blood pressure at baseline than men who converted to diabetes, compared with participants of the same sex who did not develop diabetes [40]. Correspondingly with the classic risk markers, progression from normal glucose metabolism to elevated levels of fasting glucose in women was associated with relatively greater endothelial dysfunction, a higher prevalence of hypertension, and a greater degree of dysregulated fibrinolysis and coagulation than in male counterparts [39]. Compared with men, women generally have higher fibrinolytic potential and a better endothelial function, but these protective effects are diminished in the presence of type 2 diabetes [21]. Additionally, the coagulation system is in a more pro-thrombotic state in diabetic women compared with diabetic men [21]. Finally, type 2 diabetes may induce a greater immune response and impairment of cellular defense mechanisms against oxidative stress in women than in men [41]. These sex differences in hyperglycemia-induced hemodynamics might be explained by complex interactions between insulin and estrogen signaling [42]. Whether these differences explain women’s higher relative risk on diabetes-associated cardiovascular disease requires further study.

Despite the evidence above regarding traditional risk factors, results from the meta-analyses that demonstrated that sex differences exist in the relative risk of vascular disease associated with diabetes were adjusted for traditional cardiovascular risk factors. Hence, it is conceivable that sex differences in traditional risk factor levels alone cannot fully explain the higher relative risk of women in diabetes-associated vascular disease, even though there may be unmeasured confounding. Moreover, key risk factors for vascular disease, such as total cholesterol, blood pressure, and BMI, have each been found to have a continuous log-linear association with occlusive vascular mortality in diabetic and non-diabetic individuals, which does not differ by sex [9]. Nevertheless, only baseline information about cardiovascular risk factor levels in participants with or without diabetes has been taken into account in the meta-analyses, not the possibly larger deterioration in cardiovascular risk factors levels in the conversion to diabetes. It is therefore conceivable that the risk factor changes in the conversion to diabetes explain some of the higher relative risk of vascular disease in women compared to men.

#### Future perspective

In future studies, it would be useful to investigate possible sex differences in cardiovascular risk factor levels associated with glucose metabolism status and across levels of glycemic control. Previous results from our research group indicated that there are already sex differences in cardiometabolic risk factors to women’s disadvantage before the development of type 2 diabetes, albeit weaker than in type 2 diabetes, with greater differences in systolic blood pressure and lipid levels among women than men with prediabetes and across levels of HbA1c [43]. To further understand the effects of sex differences in adiposity, detailed body composition and body fat distribution measurements conducted by DEXA and MRI can be used. These methods are appropriate to assess the extent to which fat and lean mass, visceral and subcutaneous fat, and the fat content of the liver and pancreas are differentially associated with glucose metabolism status in women and men and how such differences can explain women’s greater excess vascular disease risk associated with diabetes.

### Health care aspects

**Fig. 4:**
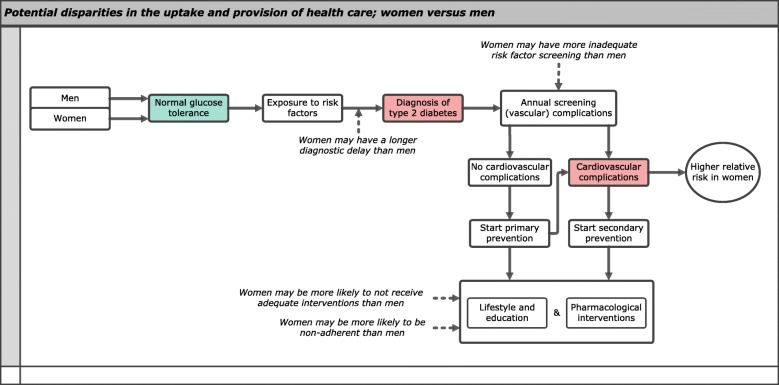
Disparities in the uptake and provision of healthcare may in part explain the excess risk of vascular disease in women with diabetes compared to their male counterparts. Potential differences in the uptake and provision of healthcare between the sexes may occur throughout the pathway—starting with healthy men and women being exposed to certain risk factors, at some point being diagnosed with diabetes and eventually developing cardiovascular complications—and may include, i.e., diagnostic delay, inadequate risk factor screening, disparities in adequate interventions, and non-adherence as shown by the arrows. The green-colored box displays normal glucose tolerance, and the red-colored boxes display negative events (i.e., type 2 diabetes, cardiovascular complications) irrespective of the sexes

In addition to sex differences in biological aspects, disparities in the uptake and provision of healthcare may in part explain sex differences in diabetes-related vascular complications (Fig. 4).

#### Diabetes management

One of the primary goals in management of diabetes is the delay and prevention of vascular morbidity and mortality [44]. Currently, many guidelines on diabetes management exist. Most of these evidence-based guidelines provide broadly similar recommendations for both sexes on diabetes management and prevention of diabetes-related complications and target lifestyle factors, including smoking behavior, physical activity, diet, and weight control, and adequate management of blood pressure, cholesterol, and glucose levels (Table 1) [3, 45].

**Table 1:** Standards of care for the management of diabetes according to the recommendations from the International Diabetes Federation

Standards of care for the management of diabetes by the International Diabetes Federation [3, 45]		
Risk factor screening	Lifestyle and education	Drug interventions and target values
Clinical assessment: - Weight, BMI, waist circumference, blood pressure, screening for retinopathy (every 1 to 2 years) and peripheral neuropathy, feet exam (every year), screening for macrovascular disease (if patient is symptomatic). Biochemical assessment: - HbA1c, lipid spectrum, renal function (every year) Lifestyle assessment: - Smoking status, overweight, physical activity, diet	Education: - Referral to diabetes education program Diet: - Reduce caloric intake with obesity or overweight, if possible referral to a dietician - Prefer high fiber and low-glycemic index foods - Avoidance of sugar, sweets, and sweetened beverages Physical activity: - Increase of physical activity Habits: - Avoid smoking - Avoid excess alcohol intake	Start lipid-lowering drugs: - T2DM and established CVD - T2DM, no established CVD, ≥ 40 years and LDL cholesterol > 100 mg/dL - T2DM, no established CVD, LDL cholesterol > 70 mg/dL may benefit especially with high 10-year CVD risk Start glucose-lowering drugs: - General HbA1c target < 7%, > 8% is generally unacceptable - HbA1c levels between 7.5 and 8% may be acceptable for patients using multiple drugs, if expected survival is limited, cognitive impairment CKD or severe CVD associated with multiple comorbidities. Start antihypertensive drugs: - Diastolic target 80 mmHg - Systolic target of 130 to 140 mmHg Start ACE-inhibitor or ARB: - Persistent albuminuria

[3,45] *CVD* cardiovascular disease, *BMI* body mass index, *T2DM* type 2 diabetes mellitus, *CKD* chronic kidney disease, *ACE-I* angiotensin converting enzyme-inhibitor, *ARB* angiotensin receptor blocker

#### Differences in health care provision

Sex differences in health care provision can broadly occur at three levels. There may be sex differences in the assessment and monitoring of vascular risk factors, in drug and lifestyle interventions for the management of risk factors, and in risk factor control among those treated. Early detection of suboptimal vascular risk factors and subsequent interventions—either lifestyle or pharmacological—significantly improves clinical outcomes [3]. Thus, any potential sex differences in the assessment or monitoring of vascular risk factors or differences in the initiation of lifestyle and/or pharmacological interventions may result in less optimal treatment, inadequate risk factor control, and consequently more severe clinical outcomes.

Two recent studies assessed sex differences in health care provision for the prevention of CHD [46, 47]. Within the general population of Australia, women were less likely to receive cardiovascular risk factor screening compared with men. However, high-risk women or women with a history of cardiovascular disease aged 65 years or older were more likely to be prescribed recommended drugs than men [46]. A large study including 10,000 individuals with coronary heart disease across Europe, Asia, and the Middle East found that risk factor management of secondary prevention was generally worse in women than men [47].

Several studies have been published on sex disparities in the management of diabetes, mainly with respect to screening of risk factors and risk factor control (Table 2). Overall, these studies have reported mixed findings regarding the presence, magnitude, and direction of sex differences in diabetes care and no definite conclusion about the impact of differences in health care provision on sex disparities in diabetes and its related cardiovascular complications can be drawn. According to most studies, women are less likely to attain risk factor control for LDL cholesterol compared with men [48–58], while risk factor control for HbA1c is more often found to be similar between sexes [49–51, 54–56, 58–61].

**Table 2:** Results from studies reporting on sex differences in screening, risk factor control, and drug interventions for diabetes

	Women do better	Women do worse	No difference between sexes	
Screening (vascular) complications				
Doctor visit	62			
BMI		44	63, 79	
(Systolic) blood pressure	44, 62	59	52, 61, 63	
Retinopathy	54, 55, 58, 62, 80	52, 57, 81, 82	79, 83	
Feet exam		44, 52, 83	57, 62	
HbA1c	58, 80	55^¶^, 56, 61**	44, 52, 53, 54, 55^‡^, 57, 59, 61^~^, 62, 63, 79, 81, 82, 83	
Lipid profile/total cholesterol/LDL cholesterol		44, 52, 53, 55^¶^, 56, 59, 61**, 81, 84	54, 57, 55^‡^, 58, 61^~^, 63, 79, 80, 82, 83	
Nephropathy		52, 55	58, 79	
Urine albumin		44, 81, 82	53, 57	
Serum creatinine	44	61**, 81	61^~^	
Smoking status	59	44, 79		
Screened for diabetes complications	63, 80	44, 59, 85	80, 82, 83	
Risk factor control				
Being on target for				
HbA1c	53, 57	50*, 51*, 52, 79, 86	49, 50, 51^†^, 54, 55, 56, 58, 59, 60, 61	
(Systolic) blood pressure	50^†^	49*, 50*, 51*, 52	49^†^, 51^†^, 57, 59, 60, 61, 79	
Total cholesterol/LDL cholesterol		48, 49*, 50, 51, 52, 53, 54, 55, 56, 57, 58, 59	49^†^, 60, 61, 79	
BMI		50, 52		
Smoking status (non-smoker)	50		59	
Being off target despite drug prescription				
Glucose-lowering drugs		52		
Lipid-lowering drugs		52		
Antihypertensive drugs			52	
Receiving drug prescription and being on target				
Glucose-lowering drugs		87	63	
Lipid-lowering drugs		63, 87		
Antihypertensive drugs	63	87*	87^†^	
Drug interventions				
Being off target and no prescription				
Glucose-lowering drugs	52			
Lipid-lowering drugs			52	
Antihypertensive drugs	52			
ACE-I or ARB		52		
Being off target and prescription				
Glucose-lowering drugs			49, 51, 63	
Lipid-lowering drugs		51*, 53	49, 51^†^, 63	
Antihypertensive drugs			49, 51, 63	
ACE-I or ARB			53	

The National Diabetes Audit – 2012–2013 studied essential care processes and achievement of treatment targets in 2 million individuals with diabetes living in England or Wales [44]. Multivariable analyses showed that women were less likely to receive assessment of all eight care processes than men and that the three recommended target levels were met by 33% and 30% of men and women, respectively. Moreover, women were less likely to receive risk factor assessment of smoking status, BMI, foot surveillance, cholesterol levels, and urine albumin and more likely to receive testing of serum creatinine and blood pressure [44]. A large population-based study from Italy, including 415,294 individuals with type 2 diabetes, demonstrated that women were less likely to receive recommended care than men [52]. In particular, women were less likely to receive assessment of kidney function and foot and eye surveillance and to achieve risk factor control of HbA1c and LDL cholesterol despite drug intervention and were more likely to have a BMI ≥ 30 than men. Women were more likely to receive insulin or antihypertensive medication than men when being off target for HbA1c or blood pressure respectively, while women were less likely to receive adequate treatment despite micro/macroalbuminuria compared to men [52]. In contrast, a large cross-sectional study among 18,000 men and women with diabetes in the US from the Medical Expenditure Panel Survey Household Component showed that, over a study period of nine years, women were more likely to receive recommended care than men [62]. In adjusted analyses, women were more likely to receive annual tests for dilated eye exams and blood pressure control and to visit a doctor; no differences were found for HbA1c testing and foot surveillance than men [62].

Although studies are inconclusive about sex differences in diabetes management, implementation of diabetes management can be improved on multiple aspects for both sexes, including assessment of risk factors and risk factor control. Rossi et al. reported that women were more likely to be off target for HbA1c and LDL cholesterol than men, despite receiving drug interventions [52]. Similar results were found in a Dutch primary care population with diabetes, showing that women receiving lipid-lowering drugs were less likely to be on target for LDL-c and more likely to attain treatment targets for blood pressure when prescribed antihypertensive drugs than men [63]. Hence, these differences in risk factor control may be caused by differences in drug type, dosage, or adherence, which is not assessed in most studies and should be investigated further.

#### Differences in drug adherence

Non-adherence to drugs is a frequent, complex, and multidimensional problem, and the World Health Organization (WHO) has described non-adherence as being “the primary reason for suboptimal benefit of therapy.” [64] Inadequate drug adherence results in suboptimal risk factor control and has been associated with adverse cardiovascular outcomes, including premature mortality [65–69]. Nonetheless, non-adherence remains difficult to define and absence of uniform research methods makes it challenging to study and reduce non-adherence [68].

Despite the major impact of non-adherence on cardiovascular outcomes, determinants including sex that drive non-adherence have not been fully identified. A large meta-analysis including 53 studies from diverse populations showed that only about 50% of men and 47% of women were adherent to statins and that women were an additional 10% more likely to be non-adherent than men [70]. Several meta-analyses and systematic reviews on non-adherence have shown that adherence rates in individuals with diabetes are also suboptimal [71–73]. Moreover, individuals with diabetes non-adherent to cardiovascular drugs were reported to have higher rates of all-cause mortality and higher hospital admission rates compared with adherent individuals [69]. Only a limited amount of studies have studied sex differences in non-adherence among individuals with diabetes, and these showed inconclusive results [74–78].

To further improve healthcare and to prevent and delay vascular complications, it is of major importance to identify sex-specific determinants that may contribute to non-adherence. Most studies on non-adherence rely on pharmacy claims refill data, self-report, pill count, or medication event monitoring systems. The disadvantage of these strategies is that none of these methods measure true medication intake. There is a need for studies that objectively measure medication adherence, which can be done by quantifying, through mass spectrometry, the presence of drug compounds in body fluids. By objectively studying non-adherence, more awareness about this complex and multidimensional problem can be generated and this may help health care providers to address this complex problem more easily.

## Perspectives and significance

Sex differences in both biological factors as in the uptake and provision of health care could contribute to women’s higher relative risk of diabetic vascular complications. While progress has been made towards understanding the underlying mechanisms, many uncertainties remain. Further research is recommended to study the impact of sex differences in biological factors and health care provision. To that end, it is important to include adequate numbers of women and men, in future studies, including in clinical trials. This could contribute to more awareness of the sex-specific risk factors of diabetic vascular complications and could eventually lead to more personalized care, including sex-specific recommendations in clinical guidelines. This will ensure that women are not affected by diabetes to a greater extent than men and will help to diminish the burden in both sexes.

## Data Availability

Not applicable
